# O12.1. EXAMINING THE NEUROBIOLOGICAL IMPACT OF CHILDHOOD TRAUMA: AN IMPORTANT ROLE FOR FRONTAL AND INSULAR REGIONS

**DOI:** 10.1093/schbul/sby015.269

**Published:** 2018-04-01

**Authors:** Marieke Begemann, Maya Schutte, Lucija Abramovic, Marco P M Boks, Neeltje Van Haren, Rene C W Mandl, Roel Ophoff, Christiaan H Vinkers, Marc Bohlken, Iris Sommer

**Affiliations:** 1 University Medical Center Utrecht; 2 University Medical Center Utrecht & Brain Center Rudolf Magnus; 3 University of California, Los Angeles; 9 University Medical Center Groningen

## Abstract

**Background:**

Childhood trauma may increase the risk for psychiatric illness by its negative impact on brain development. Studies investigating the association between childhood trauma and deviations in gray matter volume have shown inconsistent findings, often restricted by a region-of interest approach with a sole focus on the amygdala and hippocampus and without controlling for the presence of psychiatric illness.

**Methods:**

First, using a whole-brain approach in a large cross-diagnostic sample (n=554) of healthy individuals and patients with a bipolar type-I or psychotic disorder, we investigated the neurobiological correlates of childhood trauma by evaluating gray matter volume. Follow-up analyses were conducted to evaluate the effect of psychiatric illness. Second, we investigated to what extent these trauma-related structural correlates could be observed in both groups separately (healthy individuals versus patients). Participants were recruited as part of three different studies, all conducted in the University Medical Center Utrecht (the Netherlands) between 2007 and 2016. We included 554 participants: 220 healthy individuals without a psychiatric history, 250 patients with a bipolar-I disorder and 84 patients with a psychotic disorder. Childhood trauma was evaluated with the Childhood Trauma Questionnaire (CTQ-SF). Anatomical T1 MRI scans were acquired at 3T. FreeSurfer was used to assess regional brain morphology.

**Results:**

In the total sample, childhood trauma severity was associated with bilateral reductions in frontal and insular gray matter volumes. In the right hemisphere, medial orbitofrontal and superior frontal volume reductions were related to childhood trauma. These associations remained when adjusting for psychiatric illness, with the exception of the right superior frontal subregion. However, when evaluating both groups separately, these structural correlates of childhood trauma were mainly observed in patients. Healthy controls did show trauma related reductions in right medial orbitofrontal region, while this association was not significant in the patient group.

**Discussion:**

Our results suggest that gray matter reductions in the frontal and insular regions are important neurobiological correlates of childhood trauma. For future research, a whole brain approach should be applied, as cortical rather than subcortical areas may be the main correlate of childhood trauma contributing to the development of psychopathology.

